# Non-uniform polar tagging

**DOI:** 10.1186/1532-429X-16-S1-P31

**Published:** 2014-01-16

**Authors:** Vesal Moayed, Abbas N Moghaddam

**Affiliations:** 1 BME, Tehran Polytechnic, Tehran, Iran, Islamic Republic of; 2 Radiological Science, David Geffen School of Medicine at UCLA, Los Angeles, California, USA

## Background

CMR tagging in polar patterns is desirable because of its close compatibility to the annular geometry and motion of the left ventricle in short axis view. This circular symmetry does not exist in some pathological cases neither in the normal long axis view. An oval-shaped pattern is more suitable for assessment of the deformation in these conditions. Herein, we present the non-uniform polar tagging method and also present the preliminary images [1].

## Methods

Using the newly developed pulse sequence for circular tagging, we obtained the oval taglines by setting non-equal amplitudes to the gradient pairs that form the rotating excitation plane [1]. Assuming the gradient pairs as Gx & Gy, for example, in order to create vertical ovals Gx amplitude was selected greater than Gy amplitude. This pulse sequence was also used to acquire images in phantom as well as one pathological case.

## Results

**Figure 1:**
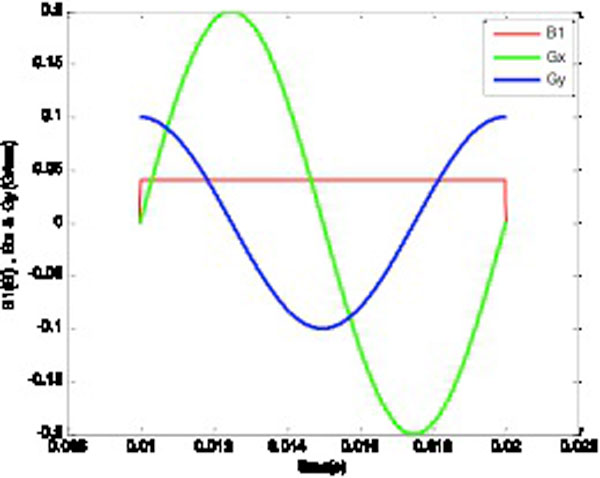
**The pulse sequence diagram for elliptical tagging**.

**Figure 2:**
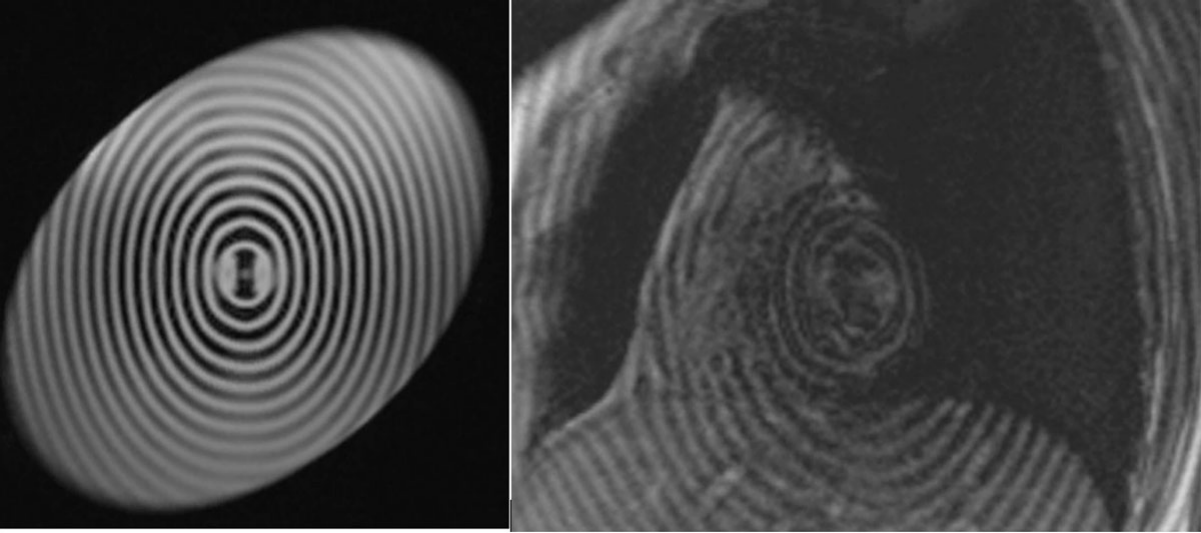
**Implemented oval shape tagging pattern on a phantom (left) and a cardiac patient (right)**.

Figure 1 illustrates the pulse sequence diagram for the proposed method for elliptical tagging with the following parameters used for simulation: Gx = 0.2 G/cm, Gy = 0.1 G/cm, B1 = 0.04 G. Figure 2 shows the corresponding images acquired from MRI scanner from both phantom and human which evidently verifies the simulation results.

## Conclusions

A pulse sequence for generating non-uniform polar tagging was theoretically described, simulated and tested in phantom and a human subject. This kind of tagging may be used to assess mayocardial deformation in a long axis view or in cases that mayocardium becomes oval-shaped in short axis view. In elliptical tagging, we can generate taglines tangent to the heart wall. The proportion of the large and small diameters of oval taglines equals to the proportion of amplitudes of the gradients, Gx and Gy. Knowing this relation we can adjust the adequate proportion for gradient amplitudes depending on the proportion of diameters of the heart so that we can lay down taglines tangent to the heart wall.

## Funding

Images were obtained through DCVI section at UCLA.
